# Immune Profiling Reveals Molecular Classification and Characteristic in Urothelial Bladder Cancer

**DOI:** 10.3389/fcell.2021.596484

**Published:** 2021-03-11

**Authors:** Li Yang, Aitian Li, Fengsen Liu, Qitai Zhao, Shaofei Ji, Wen Zhu, Weina Yu, Ru Zhang, Yaqing Liu, Wencai Li, Yi Zhang

**Affiliations:** ^1^Biotherapy Center, The First Affiliated Hospital of Zhengzhou University, Zhengzhou, China; ^2^Emergency Intervention Department, Orthopaedic Hospital of Zhengzhou City, Zhengzhou, China; ^3^Department of Urology, The First Affiliated Hospital of Zhengzhou University, Zhengzhou, China

**Keywords:** urothelial bladder cancer, immune profiling, immune cell infiltration, tumor-intrinsic signaling pathway, prognosis

## Abstract

Urothelial bladder cancer (UBC) is the most common malignant tumor of the urinary system. Most patients do not benefit from treatment with immune checkpoint inhibitors, which are closely associated with immune profiling in the context of UBC. Therefore, we aimed to characterize the immune profile of UBC to identify different immune subtypes that may influence therapy choice. We identified four subtypes of UBC based on immune profiling including immune ignorant, cold tumor, immune inactive, and hot tumor. After excluding the cold tumor subtype because of its unique pathology distinct from the other types, a high correlation between patient survival and immune characteristics was observed. Most immune cell types had highly infiltrated the hot tumor subtype compared to other subtypes. Interestingly, although immune cells infiltrated the tumor microenvironment, they exhibited an exhaustion phenotype. CCL4 may be the key molecule functioning in immune cell infiltration in the hot tumor subtype. Moreover, neutrophils may function as an important suppressor in the tumor microenvironment of the immune ignorant and immune inactive subtypes. Furthermore, different tumor-intrinsic signaling pathways were involved in immune cell infiltration and exclusion in these four different subtypes. Immune profiling could serve as a prognostic biomarker for UBC, and has potential to guide treatment decisions in UBC. Targeting tumor-intrinsic signaling pathways may be a promising strategy to treat UBC.

## Introduction

Urothelial bladder cancer (UBC) is the most common malignant tumor of the urinary system and is one of the ten most common tumors (Ferlay et al., 2015), but the treatment of UBC has seen little progress (von der Maase et al., 2005). However, some patients with advanced cancer have shown durable remission, owing to the introduction of checkpoint inhibitors and other immunotherapies (Bracarda et al., 2015; Carosella et al., 2015; Kim et al., 2015). When treated with immune checkpoint inhibitors, the objective response rate (ORR) in PD-L1^+^ bladder cancer patients was 52%, whereas the ORR in PD-L1^–^ patients with bladder cancer was only 11% (Powles et al., 2014). Therefore, most patients with UBC do not benefit from immune checkpoint inhibitors, and further study of the resistance mechanisms is needed.

The antitumor T cell response is crucial for immunotherapy, such as immune checkpoint inhibitors, to function (Harlin et al., 2009; Ji et al., 2012; Liu, 2019). In UBC, increased T cell infiltration has been found to be correlated with longer patient survival (Sharma et al., 2007). Hot tumors filled with T cells and other immune cells are often considered to be more sensitive to immunotherapy compared to cold tumors with fewer T cells; however, it is unclear why this may be the case. Variations in immune profiles have been linked to the subtypes, prognosis, and therapeutic responses of cancer (Iglesia et al., 2014; Gentles et al., 2015; Li et al., 2016). Therefore, it is critical to understand the immune profile of UBC.

Efforts have now been focused on understanding the mechanisms driving T cell exclusion and immunosuppressive cell infiltration. Immune aberrations have been shown to be closely associated with inter-tumor heterogeneity (Verhaak et al., 2010; Lehmann et al., 2011; Kim et al., 2019). Interestingly, activation of the β-catenin signaling pathway can lead to a non-T cell-inflamed phenotype in melanoma (Spranger et al., 2015). It is not yet known if tumor-intrinsic features affect the immune phenotype in UBC.

Therefore, the aim of this study was to further characterize the immune profile of UBC to identify subtypes that may be relevant for therapy choice.

## Patients and Methods

### Clinical Samples and Data Collection

A total of 2,498 immune-related genes (IRGs) were obtained from the ImmPort database^1^. RNA-seq data and clinical data from 408 UBC patients were obtained from The Cancer Genome Atlas (TCGA)^2^. All data were processed using software (version 3.6.0) or GSEA software (version 4.0.3). In addition, Molecular Signature Database (MSigDB) gene sets were downloaded from the Gene Set Enrichment Analysis (GSEA) browser^3^. Protein protein interaction (PPI) network analysis was performed based on the STRING database in http://string-db.org/cgi/input.pl.

### Identification of Immune Subtypes

Sample clustering and comparisons were performed with the R package Seurat. Transcripts per kilobase million values of UBC patients were first log-transformed and normalized, then gene expression data were analyzed by principal component analysis (PCA). The Seurat JackStrawPlot function was used to identify the number of significant principal components for each cluster. Seurat’s FindClusters function was used to generate clusters from the data, with a resolution of 0.5. We performed uniform manifold approximation and projection (UMAP) for data visualization in Seurat. Each sample yielded four clusters. The FindAllMarkers function was used to identify differentially expressed genes between the four clusters based on the following cut-off values: adjusted *P* < 0.05 and |log2 FC| > 0.5.

### Cell Infiltration and Differentially Expressed Gene Analysis

Gene expression data were used to estimate cell infiltration levels for 34 types of immune cells by R package xCell. Differentially expressed genes among subtypes were determined and estimated using the R package limma with significance criteria of adjusted *P*-value < 0.05.

### Signaling Pathway Analysis

Gene set enrichment analysis software was used to estimated significant gene ontology (GO) functions and Kyoto Encyclopedia of Genes and Genomes (KEGG) pathways enriched in each immune subtype with the significance criteria (|NES| > 1, NOM *p*-val < 0.05, FDR *q*-val < 0.25). The enrichment levels of GO functions or KEGG pathways were quantified by single-sample GSEA in the R package gsva, based on transcripts per kilobase million values of the TCGA samples. The single-sample GSEA in our study was performed with the C2 and C5 gene sets that are involved in KEGG pathways and GO functions, respectively. The C2 and C5 gene sets were retrieved from MSigDB (see text footnote 3).

### Immunohistochemistry and Immunofluorescence Staining

Human UBC tissues were obtained from The First Affiliated Hospital of Zhengzhou University in 2019, all tissues were fixed, embedded in paraffin and serially sliced. Paraffin-embedded tissue slides were dewaxed in 65°C for 1 h, hydrated in alcohol with different concentrations, heated in citrate buffer for antigen retrieval, and incubated with hydrogen peroxide for 10 min to inactivate endogenous peroxidases. Anti-human CD8 (1:200, abcam, ab199016), DSG2 (1:300, abcam, ab150372), CACNB2 antibody (1:200, abcam, ab93606) were added on slides and incubated at 4°C overnight. On the next day, slides were stained with HRP-conjunct anti-rabbit/mouse antibody (ZSGB-BIO, SP-9000). These protein expressions were visualized by DAB staining (ZSGB-BIO, ZLI-9018) following slides being counterstained with hematoxylin. The photos were recorded by microscope (PerkinElmer, Vectra).

For immunofluorescence, the sections were treated with 1% Triton X100 in 0.01 M PBS. Anti-human CD8 (1:200, abcam, ab199016), CCL4 antibody (1:100, abcam, ab45690) were added on slides and incubated at 4°C overnight. On the next day, Alexa Fluor 488-AffiniPure Donkey Anti-Mouse IgG (1:1000, Jackson, 715-545-150) and Alexa Fluor 594-AffiniPure Donkey Anti-Rabbit IgG (1:1000, Jackson, 711-585-152) were used to detect the primary antibodies. Nuclear staining was performed with DAPI (C0060, Solarbio). The stained cells were observed under microscope (PerkinElmer, Vectra), and photos were recorded.

### Statistical Analysis

The prognostic values of the four clusters, IRGs, and immune cell infiltration were estimated using the Kaplan-Meier curve and log-rank test with the R packages survival^4^ and survminer^5^. UBC samples were classified into high and low groups for IRG expression and immune cell infiltration based on the optimal cutoff value. Correlation coefficients (r) were calculated by Spearman’s correlation analysis. For comparisons of more than two groups, Kruskal–Wallis tests were used to estimate the differences. For all statistical methods, *P* < 0.05 was considered to be significantly different.

## Results

### Classifying Subtypes of UBC by Immune Profiling

**FIGURE 1:**
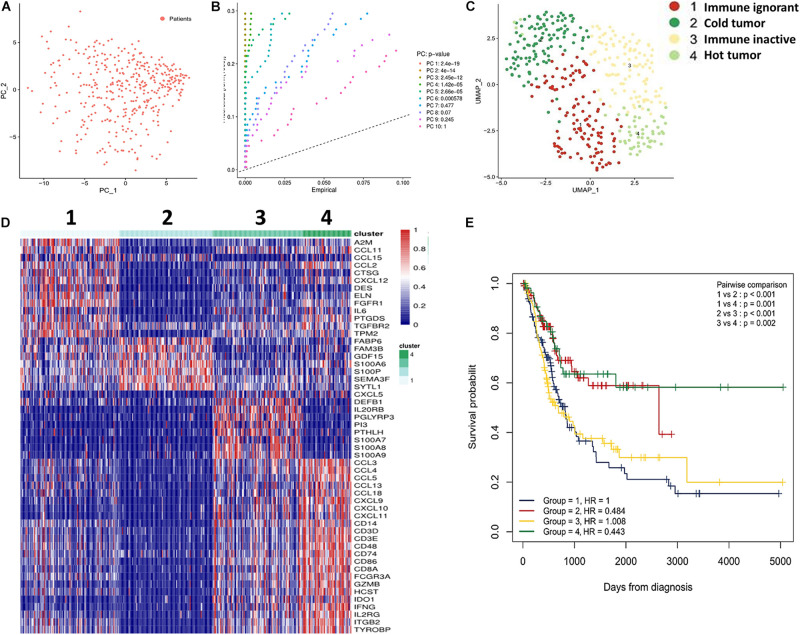
Subtypes of UBC classified by immune profiling. **(A)** IRGs (752 genes) were analyzed by PCA. **(B)** The Seurat JackStrawPlot function was used to identify the number of significant principal components for clustering. **(C)** UMAP was performed for data visualization using Seurat. **(D)** Sample clustering and comparisons were performed using the R Package Seurat. **(E)** Subtype survival was estimated by Kaplan-Meier analysis.

First, we determined whether T cell-inflamed and non-T cell-inflamed UBCs could be identified using the IRG expression signature from the Immport website (1187 genes). Next, these genes were screened by survival analysis, which revealed 752 IRGs to be significantly differentially expressed with *P* < 0.05. Furthermore, these 752 genes were analyzed using PCA (Figure 1A). In total, ten principal components were identified, and, among them, six principal components had significant differences with *P* < 0.05 (Figure 1B). These six principal components were then analyzed using UMAP (Figure 1C). The results identified four subtypes: immune ignorant, cold tumor, immune inactive, and hot tumor (subtype 1–4, Figure 1D). Among these subtypes, tumors with an immunosuppressive phenotype were defined as immune ignorant (subtype 1, *n* = 117), tumors that were completely devoid of immune infiltration were defined as a so-called cold tumor (subtype 2, *n* = 110), tumors that lack activation of the interferon signaling pathway and have no response to immunotherapy were categorized as immune inactive (subtype 3, *n* = 107), and tumors infiltrated by T cells and other immune cells were referred to as a hot tumor (subtype 4, *n* = 57). Overall survival (OS) analysis revealed that UBC patients with tumors belonging to different subtypes exhibited differential survival (Figure 1E). Patient survival in subtypes 2 and 4 was better than that of subtypes 1 and 3 (Figure 1E). Therefore, UBCs could be grouped based on their expression of genes indicative of an immune cell-inflamed tumor microenvironment.

We further evaluated the relationship between populations of the four subtypes and clinical pathological parameters. The results indicated that the pathology of most patients in the cold tumor subtype (subtype 2) were papillary, whereas other subtypes were primarily non-papillary (Supplementary Figure 1A). Moreover, other clinical parameters, including grade, clinical stage, tumor (T) status, and lymph node (N) status, of patients in the cold tumor subtype were significantly different compared to patients in other subtypes (Supplementary Figures 1B–E), indicating that patients in the cold tumor subtype were in the early stage of the tumor. This explains why patients in the cold tumor subtype exhibited good survival.

Next, we analyzed the correlation between the expression of T cell signature genes (CCL4, CCL5, CCL8, CCL13, CCL18, CCR5, CD3D, CD3E, CD4, and CD8A) and patient survival. For this analysis, patients in the cold tumor subtype were excluded due to their unique pathological type and early clinical stage. The results revealed that low expression levels of these genes were associated with poor OS (Supplementary Figure 2). These data demonstrate that the T cell signature genes could serve as potential prognostic biomarkers.

### Characteristics of Immune Cell Infiltration in UBC

To further investigate and confirm the immune cell infiltration in different subtypes of UBC, we analyzed and estimated the following 34 subpopulations of immune cells: activated T cells, including CD4^+^ and CD8^+^ naïve T cells, central memory T cells (Tcm) and effector memory T cells (Tem), gamma delta T (Tgd) cells, T helper 1 (Th1) cells, Th2 cells, regulatory T cells (Tregs); naïve, class-switched memory, memory, and pro-B cells; along with cell subtypes related to innate immunity, such as monocytes, M1/M2-like macrophages, mast cells, eosinophils, neutrophils, plasmacytoid, activated, and conventional, immature dendritic cells (pDC, aDC, cDC, and iDC), NK cells, and natural killer T (NKT) cells.

**FIGURE 2:**
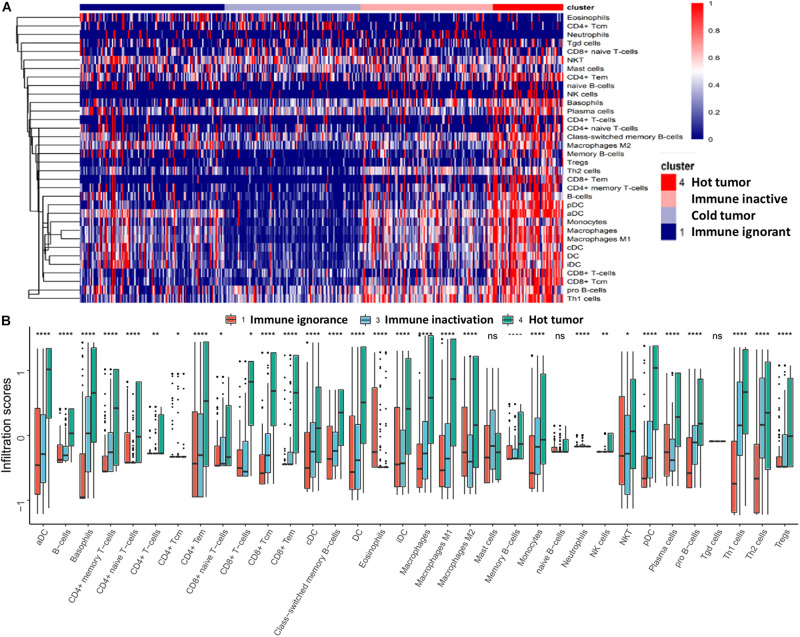
Characteristics of immune cell infiltration in UBC. **(A)** Immune cell infiltration in the four subtypes is presented as a heatmap. **(B)** Comparison of immune cell infiltration in different subtypes. Data are shown as boxplots. *P < 0.05, **P < 0.01, ****P < 0.0001, ns, non-significant.

We observed heterogeneity across these four subtypes of UBC. The hot tumor subtype (subtype 4) was enriched for immune cell infiltration; however, the other three subtypes were lacking immune cell infiltration (Figure 2A). Excluding the cold tumor subtype, we further compared the degree of immune cell infiltration between the other three subtypes. The results revealed that most immune cells had highly infiltrated the hot tumor subtype compared with the other subtypes (Figure 2B). These findings suggest that patients in the hot tumor subtype have a high degree of immune cell infiltration and have good antitumor immunity, which may explain why patients in the hot tumor subtype exhibited good survival.

Additionally, the correlation between immune cells and patient survival was further analyzed. We found that immune cell profiling was positively associated with survival, high levels of immune cell infiltration, and specifically related to adaptive immunity, including CD4 + T cells, Tcm and Tem CD4 + and CD8 + T cells, naïve CD8 + T cells, cDC, DC, and class switched memory B cells, resulting in a good prognosis (Supplementary Figure 3). In summary, immune cell profiling could function as a prognostic biomarker for UBC.

### Activated Immune Cells Exhibit an Immunosuppressive Phenotype

**FIGURE 3:**
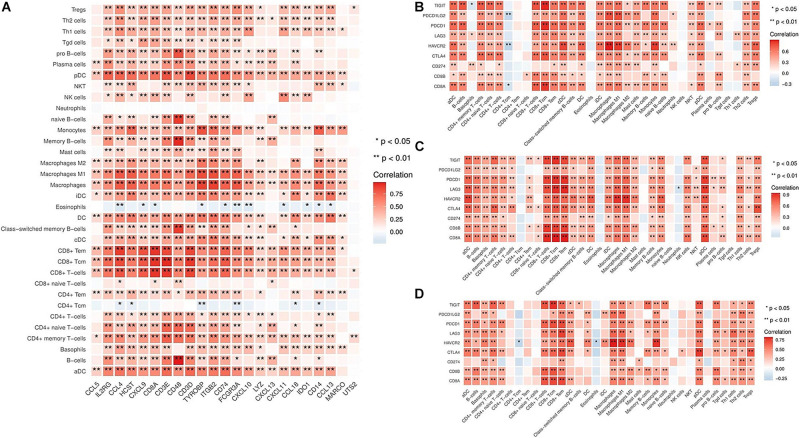
Correlation between immune cell infiltration and IRG expression in different subtypes. **(A)** The relationship between immune cell infiltration and IRG expression in the hot tumor subtype. **(B)** The relationship between immune cell infiltration and inhibitory molecules (TIGIT, PDCD1LG2, PDCD1, LAG3, HAVCR2, CTLA4, and CD274) in the immune ignorant subtype. **(C)** The relationship between immune cell infiltration and inhibitory molecules in the immune inactive subtype. **(D)** The relationship between immune cell infiltration and inhibitory molecules in the hot tumor subtype. *P < 0.05, **P < 0.01.

Based on the above results, we identified the hot tumor subtype as an immune cell-inflamed tumor, particularly a T cell-inflamed tumor. Moreover, the expression of IRGs, including CD8A, CXCL9, CXCL10, CXCL11, CCL4, CCL5, CCL13, CCL18, IL2RG, etc., were markedly higher in the hot tumor subtype compared to the other subtypes (Supplementary Figure 4). Next, we investigated the correlation between the immune-related molecules highly expressed in the hot tumor subtype and immune cell infiltration in the tumor microenvironment. The results showed that the expression of IRGs was closely correlated with immune cell infiltration (Figure 3A), revealing that high expression of IRGs, especially of chemokines that recruit T cells, such as CXCL9, CXCL10, CXCL11, etc., could induce an increase in immune cell infiltration in bladder cancers of the hot tumor subtype.

It has been shown that increased levels of immune inhibitory molecules are associated with a T cell-inflamed phenotype (Spranger et al., 2013). Therefore, we evaluated the relationship between the expression of immune inhibitory molecules (TIGIT, PDCD1LG2, PDCD1, LAG3, HAVCR2, CTLA4, and CD274) in the immune ignorant, immune inactive, and hot tumor subtypes. This revealed that inhibitory molecules were significantly more highly expressed in immune cells (Figures 3B–D). Furthermore, the expression of these inhibitory molecules was positively correlated with the T cell signature gene CD8A (Supplementary Figure 5). Interestingly, although immune cells had infiltrated the tumor microenvironment, they exhibited an exhaustion phenotype.

### Enriched Signaling Pathways Involved in T Cell Infiltration in the Hot Tumor Subtype

**FIGURE 4:**
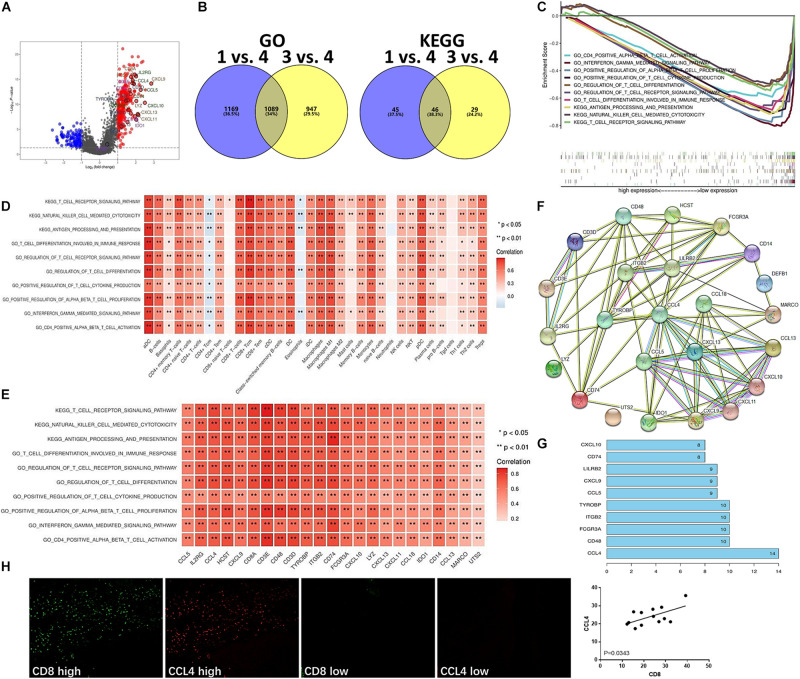
Enriched signaling pathways involved in T cell infiltration in the hot tumor subtype. **(A)** Differentially expressed genes in the hot tumor subtype versus other subtypes (4 vs. 1 and 3). **(B)** Signaling pathway enrichment in different subtypes (1 vs. 4, and 3 vs. 4) was analyzed using GO and KEGG analysis. **(C)** Enriched signaling pathways in the hot tumor subtype were analyzed and are presented. **(D)** Correlation between enriched signaling pathways and immune cell infiltration in the hot tumor subtype. **(E)** Correlation of enriched signaling pathways and IRGs in the hot tumor subtype. **(F)** Network of the high level immune-related molecules in the hot tumor subtype. **(G)** The number of molecules linked with one another in the hot tumor subtype was analyzed. **(H)** CCL4 and CD8 expression in human UBC tissues was analyzed by immunofluorescence. Data are presented as a bar chart. *P < 0.05, **P < 0.01.

To validate which signaling pathways were involved in immune cell infiltration, especially in the T cell-inflamed tumor microenvironment, we further categorized two different gene populations with high and low expression in the hot tumor subtype (Figure 4A). Then, we assessed signaling pathway enrichment by comparing the immune ignorant versus hot tumor subtype (1 vs. 4) and the immune inactive versus hot tumor subtype (3 vs. 4). GO enrichment results revealed that 1,089 signaling pathways were screened, and 46 KEGG pathways were found to be enriched (Figure 4B). Based on these enriched signaling pathways, ten signaling pathways with significant standard that survival analyses were selected and included CD4 positive alpha beta T cell activation, interferon gamma mediated signaling pathway, positive regulation of alpha beta T cell proliferation, positive regulation of T cell cytokine production, regulation of T cell differentiation, regulation of T cell receptor signaling pathway, T cell differentiation involved in immune response, antigen processing and presentation, natural killer cell mediated cytotoxicity, and T cell receptor signaling pathway (Figure 4C). To evaluate whether the high level of immune cell infiltration was mediated by these enriched signaling pathways, their correlation was analyzed. The results indicated that these enriched signaling pathways were positively correlated with increased infiltration of most immune cell types (Figure 4D), and with high expression of IRGs in the hot tumor subtype (Figure 4E). The above data has revealed that the regulation of the T cell differentiation and activation signaling pathways are involved in immune cell infiltration in the hot tumor subtype.

Next, we investigated the interaction of these high level immune-related molecules in the hot tumor subtype and found a strong interaction between these molecules (Figure 4F). Additionally, CCL4 was the most connected with other molecules (Figure 4G), suggesting that CCL4 could be the key molecule functioning in the immune cell infiltration of the hot tumor subtype. Moreover, immunofluorescence results showed that CCL4 level was positively correlated with CD8 level in human UBC tissues (Figure 4H), indicating that CD8^+^ T cell infiltration is closely associated with CCL4 in the hot tumor subtype.

### Enriched Signaling Pathways Involved in the Lack of Immune Cell Infiltration in the Immune Ignorant and Immune Inactive Subtypes

**FIGURE 5:**
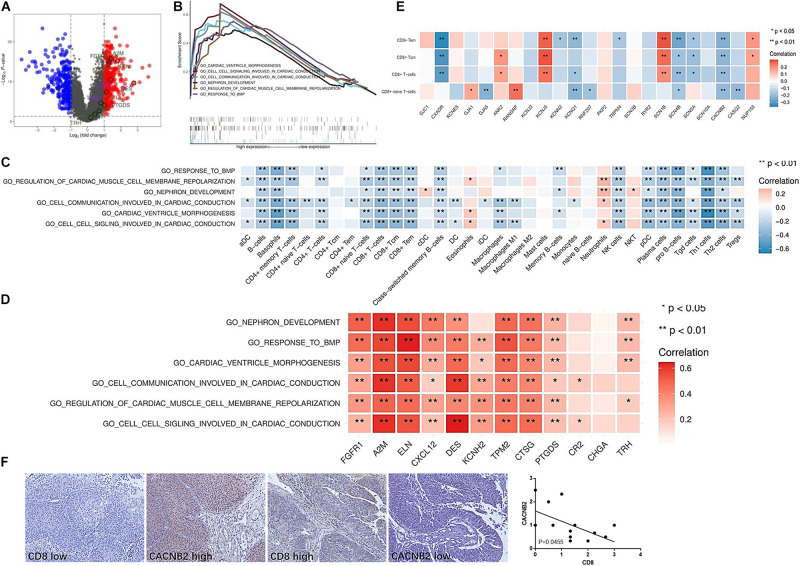
Enriched signaling pathways involved in the lack of immune cell infiltration in the immune ignorant subtype. **(A)** Differentially expressed genes in the immune ignorant subtype with respect to other subtypes. **(B)** Enriched signaling pathways in the immune ignorant subtype were analyzed and are presented. **(C)** Correlation between enriched signaling pathways and immune cell infiltration in the immune ignorant subtype. **(D)** Correlation between enriched signaling pathways and IRGs in the immune ignorant subtype. **(E)** Correlation between cell signaling involved in cardiac conduction signaling pathway-related molecules and CD8^+^ T cell infiltration in the immune ignorant subtype. **(F)** CACNB2 and CD8 expression in human UBC tissues was analyzed by immunohistochemistry. *P < 0.05, **P < 0.01.

Tumor-intrinsic signaling pathway has been reported to mediate the immune phenotype of the tumor microenvironment. To develop new therapeutic strategies that improve the response to immunotherapies, it has been a priority to identify the molecular signaling pathways that function in tumor cells and may capable of inducing the exclusion of immune cells, especially T cells, from the tumor microenvironment. Therefore, we hypothesized that some tumor-intrinsic signaling pathways may be involved in the exclusion of immune cells in the immune ignorant and immune inactive subtypes. First, two different gene populations were grouped based on high and low expression in the immune ignorant subtype (Figure 5A). Additionally, five signaling pathways that were highly expressed and had significant survival analyses were identified and included including cardiac ventricle morphogenesis, cell-cell signaling involved in cardiac conduction, cell communication involved in cardiac conduction, nephron development, regulation of cardiac muscle cell membrane repolarization, and response to BMP signaling pathway (Figure 5B). Correlation analysis revealed that these signaling pathways were negatively associated with decreased immune cell infiltration, particularly T cell exclusion (Figure 5C), and positively associated with the expression level of IRGs, which were highly expressed in the immune ignorant subtype (Figure 5D). Finally, the related genes (CXADR, KCNQ1, SCN4B, and CACNB2) of the cell-cell signaling involved in cardiac conduction signaling pathway, the most enriched pathway in the immune ignorant subtype, were negatively associated with CD8^+^ T cell infiltration (Figure 5E). Furthermore, we analyzed the correlation between CD8 and CACNB2 by immunohistochemistry, showing that CACNB2 level was negatively correlated with CD8 level in human UBC tissues (Figure 5F). Therefore, the lack of T cells in the tumor microenvironment could be mediated by cell-cell signaling involved in cardiac conduction signaling pathway in the immune ignorant subtype.

**FIGURE 6:**
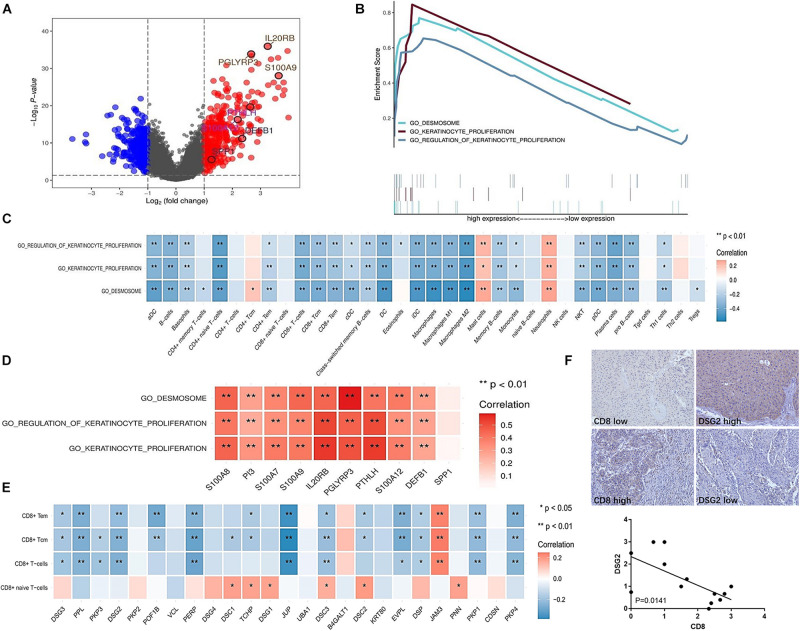
Enriched signaling pathways involved in the lack of immune cell infiltration in the immune inactive subtype. **(A)** Differentially expressed genes in the immune inactive subtype with respect to other subtypes. **(B)** Enriched signaling pathways in the immune inactive subtype were analyzed and are presented. **(C)** Correlation between enriched signaling pathways and immune cell infiltration in the immune inactive subtype. **(D)** Correlation between enriched signaling pathways and IRGs in the immune inactive subtype. **(E)** Correlation between desmosome signaling pathway-related molecules and CD8^+^ T cell infiltration in the immune inactive subtype. **(F)** DSG2 and CD8 expression in human UBC tissues was analyzed by immunohistochemistry. *P < 0.05, **P < 0.01.

Additionally, the mechanism underlying T cell exclusion from the tumor microenvironment in the immune inactive subtype was identified and analyzed. The significantly different genes were divided into two groups (Figure 6A). Three main signaling pathways were enriched in the immune inactive subtype, including desmosome, keratinocyte proliferation, and regulation of keratinocyte proliferation signaling pathway (Figure 6B), and these three pathways also had a strong survival significance. Correlation analysis revealed that these three signaling pathways were negatively correlated with the lack of immune cell infiltration, especially T cell exclusion (Figure 6C), and were also positively correlated with the levels of IRGs, which were highly expressed in the immune inactive subtype (Figure 6D). The related genes (PPL, DSG2, PERP, and JUP, etc.) of the desmosome signaling pathway, the most enriched pathway in the immune inactive subtype, were negatively associated with CD8^+^ T cell infiltration (Figure 6E). And we also found that DSG2 level was negatively correlated with CD8 level in human UBC tissues (Figure 6F). Taken together, these results indicate that tumor-intrinsic signaling pathways are involved in the decrease of immune cell infiltration in the non-T cell-inflamed phenotype.

Immune profiling in the immune inactive subtype was similar to that of the immune ignorant subtype, demonstrating that neutrophils were increased in the tumor microenvironment via the crucial signaling pathways found in both the immune ignorant and immune inactive subtypes. Neutrophils were found to function as an important suppressor, in opposition of antitumor immunity, in the tumor microenvironment of the immune ignorant and immune inactive subtypes.

## Discussion

The classification of bladder cancer is dependent on various types of profiling. Muscle-invasive bladder cancer patients treated with bladder-sparing trimodality therapy have been classified into luminal, luminal-infiltrated, basal, and claudin-low subtypes based on whole transcriptome expression profiling (Efstathiou et al., 2019). Sweis et al. (2016) analyzed 267 samples of UBC from a TCGA dataset and divided into the samples into T cell-inflamed and non-T cell-inflamed subtypes based on immune gene profiling. A cluster of 725 genes containing 12 T cell signature genes was used to perform consensus clustering of tumor samples. In the current study, we used 752 IRGs to classify subtypes of UBC. The results identified the following four subtypes: immune ignorant, cold tumor, immune inactive, and hot tumor. Among these subtypes, subtype 1 is defined as immune ignorant and presents an immunosuppressive phenotype and decreased immune cell infiltration; subtype 2 is defined as cold tumor due to the complete lack of immune cell infiltration; subtype 3 is referred to as immune inactive due to the lack of active interferon signaling and failure to respond to immunotherapy; subtype 4 is defined as hot tumor due to the enrichment of T cells and other immune cells in the tumor microenvironment.

Among these four subtypes, the cold tumor subtype is unique (Fang et al., 2018). The results indicated that the clinical pathology of patients in the cold tumor subtype was primarily papillary, whereas other subtypes were primarily non-papillary. Moreover, other clinical parameters, including grade and clinical stage, T and N statuses indicated that patients in the cold tumor subtype were in the early stage of UBC. This explains why patients in the cold tumor subtype exhibited good survival given the unique clinical pathology compared to other subtypes.

A variety of papers have demonstrated a close relationship between immunity and patient survival. DC-SIGN + macrophages were correlated with poor prognosis and inferior therapeutic response to fluorouracil-based adjuvant chemotherapy, and may serve as an independent prognostic factor for gastric cancer (Liu et al., 2020). In addition, intratumoral CD8 status had an obvious effect on prognosis. In patients with high levels of intratumoral CD8, PD-L1 expression revealed no significant prognostic impact; however, in patients with low levels of intratumoral CD8, the presence of PD-L1 was associated with a significantly worse prognosis compared to the control (Handa et al., 2020). Moreover, Sun et al. (2018) developed a radiomic signature for CD8 cells, and a high baseline radiomic score was correlated with improved OS. In our study, we also used IRGs as prognostic biomarkers in UBC. High levels of T cell signature genes (CCL4, CCL5, CCL8, CCL13, CCL18, CCR5, CD3D, CD3E, CD4, and CD8A) were associated with a good OS. Furthermore, we found that a high level antitumor immune cell infiltration was positively associated with survival, and provided a good prognosis, suggesting that immune cell profiling could serve as a prognostic biomarker for UBC.

In the current study, we also found that cancer patients within the four subtypes exhibited different survivals. Excluding the cold tumor subtype (the unique subtype with an early clinical stage), patients in the hot tumor subtype with high immune infiltration exhibited good antitumor immunity, which may explain why patients in the hot tumor subtype demonstrated better survival. In other published papers, the immune active subtype has been associated with improved survival and shared similar genomic characteristics with those who responded to anti-PD-1 therapy (Zhou et al., 2020). In addition, the immunosuppressive subtype was found to feature high immune infiltration, stromal enrichment, and activation of the transforming growth factor (TGF)-β signaling pathway was correlated with the non-responsive signature of immune checkpoint inhibitor therapy, which may be required for the combination therapy of anti-PD-L1 and anti-TGF-β (Zhou et al., 2020).

T cell exhaustion is a hallmark of cancers and is characterized by the increase of several immune checkpoints that lead to the failure of immune checkpoint inhibitors. Woroniecka et al. (2018) observed the poor function of infiltrated T cells in the tumor microenvironment of glioblastoma. In localized clear cell renal cell carcinoma, the intratumoral infiltration of exhausted CD8^+^ T cells with high levels of PD-1, Tim-3, and Lag-3 was investigated (Giraldo et al., 2017). The current study also found that although immune cells had infiltrated the tumor microenvironment of UBC, immune cells, especially T cells, exhibited an exhaustion phenotype.

In our previous study, the activation of specific tumor-intrinsic signaling pathways could be explained the phenomenon of immune exclusion in a subset of cancers (Yang et al., 2019). Increasing evidence has indicated that tumor-intrinsic signaling plays an important role in regulating tumor immune escape. Activation of the β-catenin signaling pathway within melanoma tumor cells excludes immune cell activation and leads to a non-T cell-inflamed tumor microenvironment (Spranger et al., 2015). Constitutively active STAT3 signaling in tumor cells has been shown to downregulate chemokine expression, including CCL5 and CXCL10, which are functionally responsible for T cell recruitment (Wang et al., 2004; Burdelya et al., 2005). Profiling the expression of IRGs identified three molecular pathways linked with the non-T cell-inflamed subtype (Fang et al., 2018). In this study, the tumor-intrinsic signaling pathways, such as cardiac ventricle morphogenesis, cell communication involved in cardiac conduction, nephron development, regulation of cardiac muscle cell membrane repolarization, and response to BMP signaling pathway, may be involved in the non-T inflamed tumor microenvironment of the immune ignorant subtype. Moreover, desmosome, keratinocyte proliferation, and regulation of keratinocyte proliferation signaling pathway may be responsible for T cell exclusion in the immune inactive subtype.

In our study, we observed a relatively increase of neutrophils in the immune inactive subtype and immune ignorant subtype, which were found to function in opposition of antitumor immunity. Mandelli et al. (2020) reported that basal type of bladder cancer contained a significantly higher density of CD66b^+^ tumor-associated neutrophils (TANs) compared to the luminal type, and a high density of TANs and T cells was significantly associated with a better outcome. However, Liu et al. (2018) found that elevated CD66b^+^ TAN was correlated with an advanced T-stage, a high grade, a worse recurrence-free survival within non-muscle invasive bladder cancer subgroup and a worse overall survival within all urothelial bladder cancer cases. Therefore, Neutrophils might show different phenotypes and roles in different histological types of bladder cancers.

## Conclusion

Urothelial bladder cancer can be classified into four subtypes by immune profiling. The hot tumor subtype has a high level of immune cell infiltration and is closely associated with good patient survival. IRGs and infiltrated immune cells can serve as potential biomarkers for prognosis. Tumor-intrinsic signaling pathways may play a key role in intratumoral T cell exclusion and poor prognosis in the immune ignorant and immune inactive subtypes. Therefore, targeting these signaling pathways represents a promising strategy for the treatment of UBC.

## Data Availability Statement

The original contributions presented in the study are included in the article/Supplementary Material, further inquiries can be directed to the corresponding author/s.

## Author Contributions

LY and YZ designed, edited, and led out this study. AL, FL, QZ, SJ, WZ, WY, and RZ conducted the data analysis and critical discussions of the results. LY, AL, FL, QZ, YL, and WL provided material support and study supervision. All authors contributed to the writing and editing of the manuscript, and approved the final draft of the manuscript.

## Conflict of Interest

The authors declare that the research was conducted in the absence of any commercial or financial relationships that could be construed as a potential conflict of interest.

## Footnotes

1 https://www.immport.org/home

2 https://portal.gdc.cancer.gov/

3 https://www.gsea-msigdb.org/gsea/index.jsp

4 https://cran.r-project.org/package=survival

5 https://cran.r-project.org/package=survminer

## Supplementary Material

Supplementary Figure 1 Clinical pathologies of the four subtypes. **(A)** The percent of papillary and non-papillary pathology observed in subtypes 1–4. **(B)** The percent of high and low grade observed in subtypes 1–4. **(C)** The percent of stage I-IV observed in subtypes 1–4. **(D)** The percent of T1-T4 observed in subtypes 1–4. **(E)** The percent of N1-N4 observed in subtypes 1–4.

Supplementary Figure 2 The effect of IRGs on patient survival. Kaplan-Meier survival curves for UBC patients with high and low expression of IRGs.

Supplementary Figure 3 The effect of immune cell infiltration on patient survival. Kaplan-Meier survival curves for UBC patients with high and low levels of immune cell infiltration.

Supplementary Figure 4 Comparison of IRG expression in the immune ignorant, immune inactive, and hot tumor subtypes. IRGs included CCL13, CCL18, CCL4, CCL5, CD14, CD3D, CD3E, CD48, CD74, CD8A, CXCL10, CXCL11, CXCL13, CXCL9, FCGR3A, HCST IDO1, IL2RG, ITGB2, LYZ, MARCO, TYROBP, and UTS2. Data are shown as boxplots. *****P* < 0.0001.

Supplementary Figure 5 Correlation between CD8A and inhibitory molecules in UBC. Inhibitory molecules included PD-L1, TIM3, LAG3, CD163, CD39, CD73, IDO, IL-10, TGFB1I1, TGFB2, TGFB3, and IL6. Correlation coefficient (r) was calculated by Pearson’s correlation analysis.

## Abbreviations

UBC: urothelial bladder cancer
ORR: objective response rate
IRGs: immune-related genes
TCGA: The Cancer Genome Atlas
MSigDB: Molecular Signature Database
GSEA: Gene Set Enrichment Analysis
PPI: Protein protein interaction
PCA: principal component analysis
UMAP: uniform manifold approximation and projection
GO: gene ontology
KEGG: Kyoto Encyclopedia of Genes and Genomes
OS: overall survival
Tcm: central memory T cells
Tem: effector memory T cells
Tgd: gamma delta T
Th1: T helper 1
Th2: T helper 1
Tregs: regulatory T cells
pDC: plasmacytoid dendritic cells
aDC: activated dendritic cells
cDC: conventional dendritic cells
iDC: immature dendritic cells
NKT: natural killer T cell
TGF: transforming growth factor.
