# Supplementary figures and images for: Immune Profiling Reveals Molecular Classification and Characteristic in Urothelial Bladder Cancer
Source: Front Cell Dev Biol. 2021 Mar 11;9:596484. doi: 10.3389/fcell.2021.596484 (PMC7990773; doi:10.3389/fcell.2021.596484)

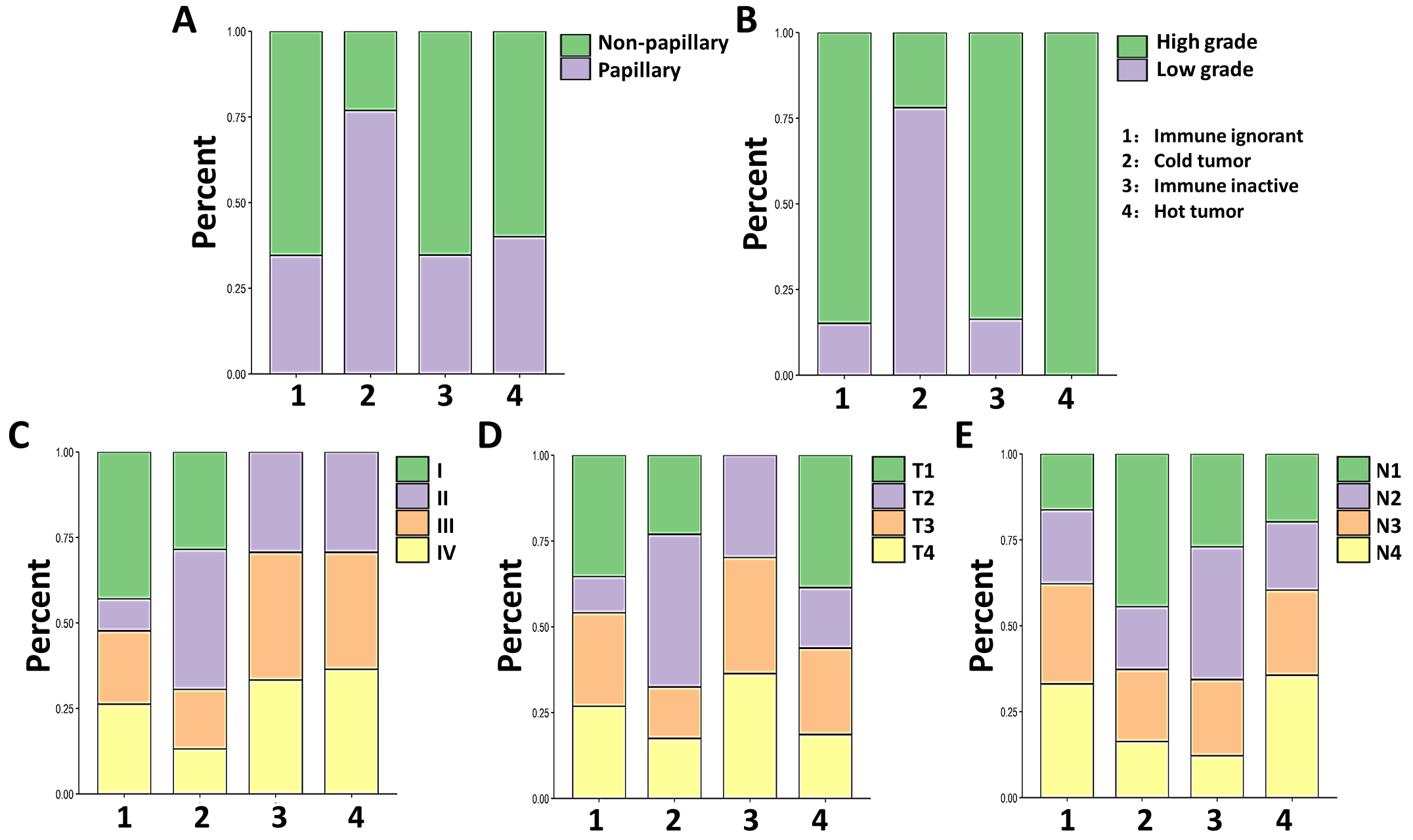

Supplement: Supplementary Figure 1 — Clinical pathologies of the four subtypes. (A) The percent of papillary and non-papillary pathology observed in subtypes 1–4. (B) The percent of high and low grade observed in subtypes 1–4. (C) The percent of stage I-IV observed in subtypes 1–4. (D) The percent of T1-T4 observed in subtypes 1–4. (E) The percent of N1-N4 observed in subtypes 1–4. [file Image_1.TIF]

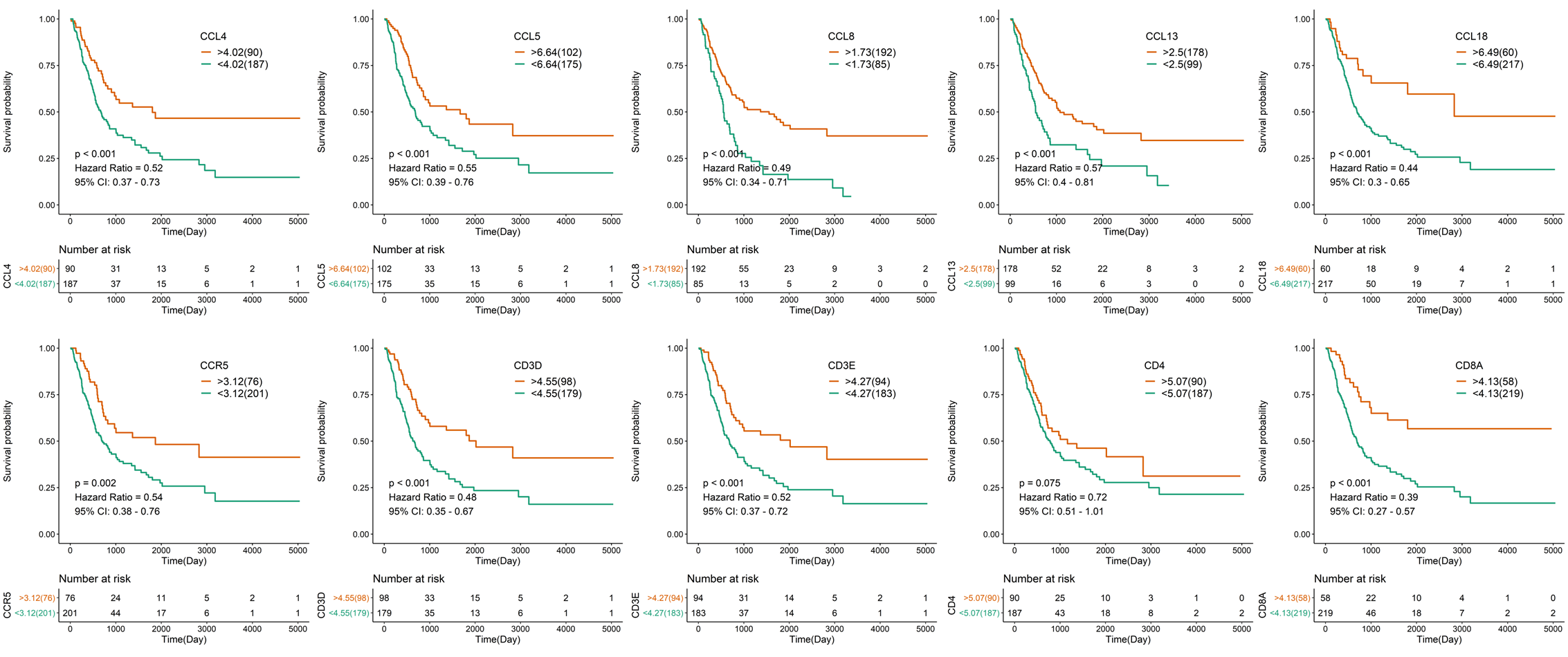

Supplement: Supplementary Figure 2 — The effect of IRGs on patient survival. Kaplan-Meier survival curves for UBC patients with high and low expression of IRGs. [file Image_2.TIF]

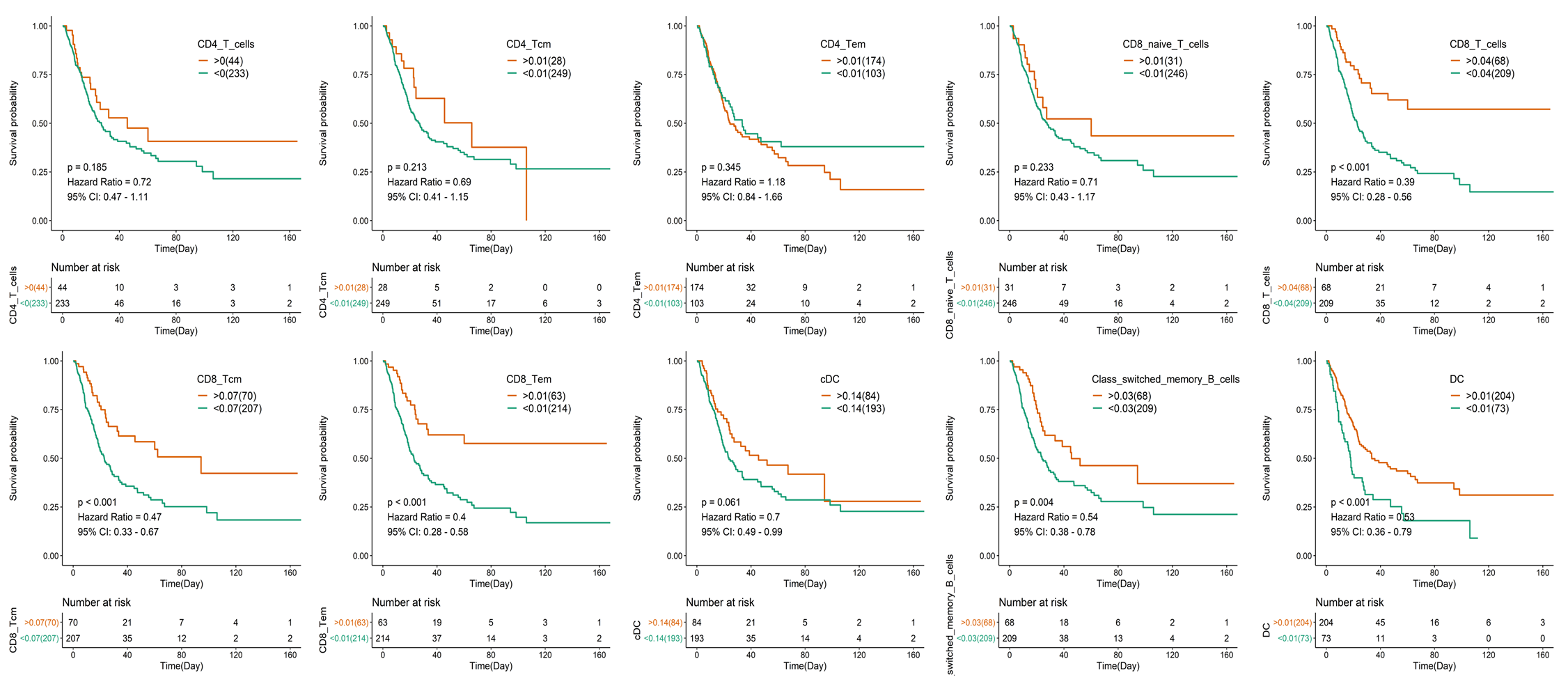

Supplement: Supplementary Figure 3 — The effect of immune cell infiltration on patient survival. Kaplan-Meier survival curves for UBC patients with high and low levels of immune cell infiltration. [file Image_3.TIF]

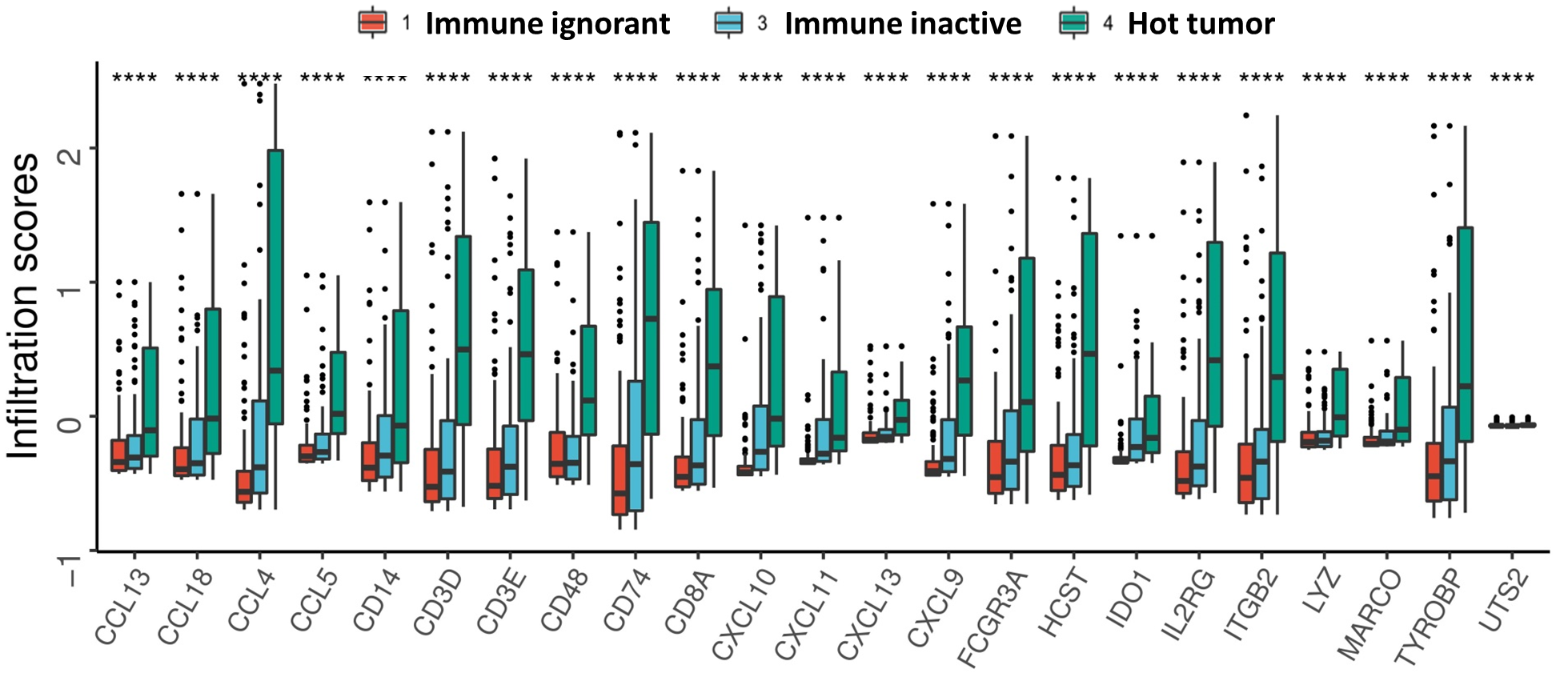

Supplement: Supplementary Figure 4 — Comparison of IRG expression in the immune ignorant, immune inactive, and hot tumor subtypes. IRGs included CCL13, CCL18, CCL4, CCL5, CD14, CD3D, CD3E, CD48, CD74, CD8A, CXCL10, CXCL11, CXCL13, CXCL9, FCGR3A, HCST IDO1, IL2RG, ITGB2, LYZ, MARCO, TYROBP, and UTS2. Data are shown as boxplots. ****P < 0.0001. [file Image_4.TIF]

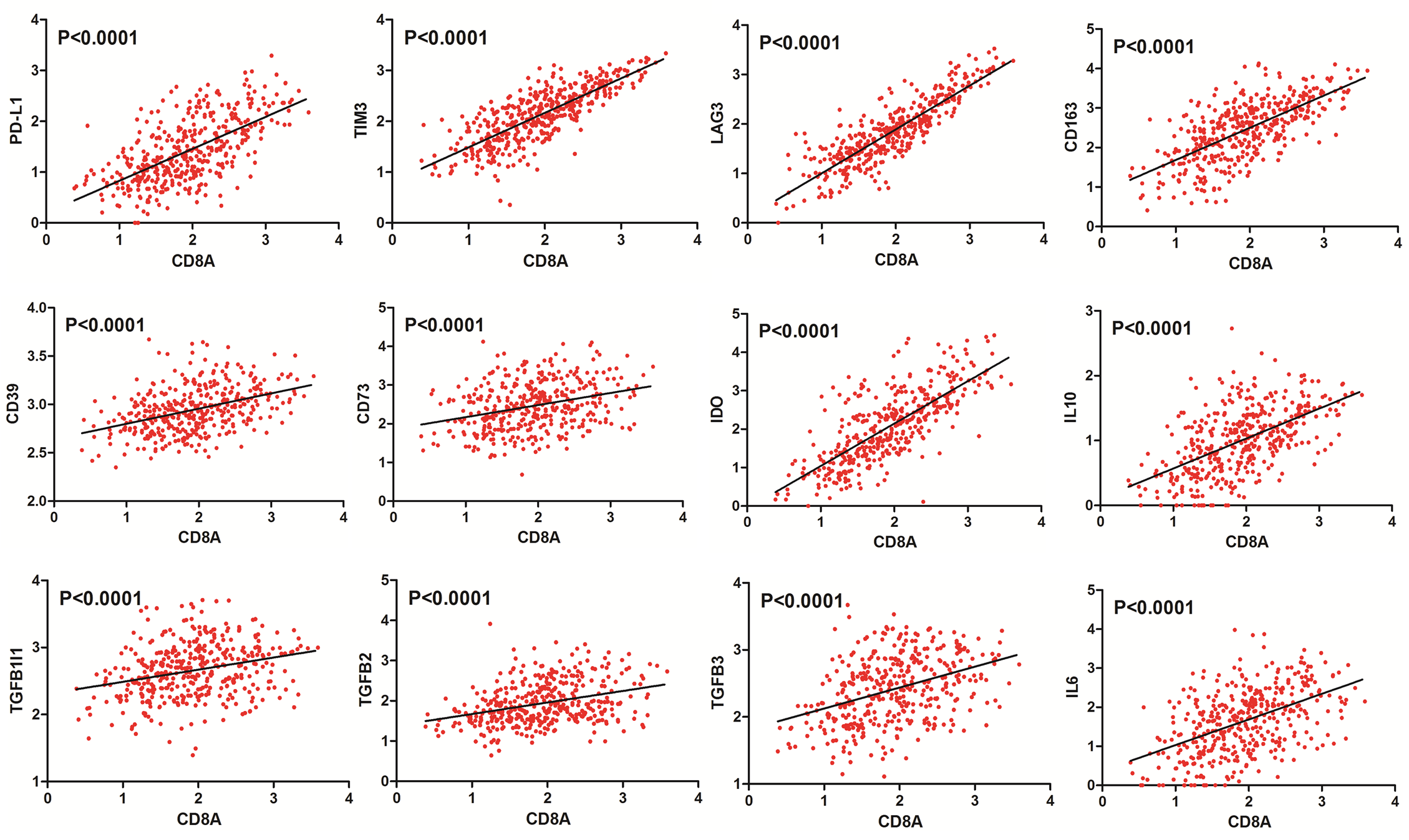

Supplement: Supplementary Figure 5 — Correlation between CD8A and inhibitory molecules in UBC. Inhibitory molecules included PD-L1, TIM3, LAG3, CD163, CD39, CD73, IDO, IL-10, TGFB1I1, TGFB2, TGFB3, and IL6. Correlation coefficient (r) was calculated by Pearson’s correlation analysis. [file Image_5.TIF]
